# Impact of Anti-Inflammatory Drugs on Pyogenic Vertebral Osteomyelitis: A Prospective Cohort Study

**DOI:** 10.1155/2016/9345467

**Published:** 2016-10-19

**Authors:** Aurélien Dinh, Maxime Jean, Frédérique Bouchand, Benjamin Davido, Alexis Descatha, Clara Duran, Guillaume Gras, Christian Perronne, Denis Mulleman, Jérôme Salomon, Louis Bernard

**Affiliations:** ^1^Infectious Diseases Unit, University Hospital R. Poincaré, APHP, Versailles Saint Quentin University, Garches, France; ^2^Pharmacy Department, University Hospital R. Poincaré, APHP, Versailles Saint Quentin University, Garches, France; ^3^Infectious Diseases Unit, University Hospital of Bretonneau, Denis Diderot University, Tours, France; ^4^Rheumatology Department, University Hospital of Bretonneau, Denis Diderot University, Tours, France

## Abstract

*Objective*. Pyogenic vertebral osteomyelitis (PVO) are frequently misdiagnosed and patients often receive anti-inflammatory drugs for their back pain. We studied the impact of these medications.* Methods*. We performed a prospective study enrolling patients with PVO and categorized them depending on their drugs intake. Then, we compared diagnosis delay, clinical presentation at hospitalization, incidence of complications, and cure rate.* Results*. In total, 79 patients were included. Multivariate analysis found no correlation between anti-inflammatory drug intake and diagnosis delay, clinical presentation, complications, or outcome.* Conclusion*. Anti-inflammatory drugs intake does not affect diagnostic delay, severity at diagnosis, or complications of PVO.

## 1. Introduction

Pyogenic vertebral osteomyelitis (PVO) is a rare disease: its incidence is estimated at 4 to 10 per 100 000 inhabitants per year in high-income countries [1, 2] and has risen in recent years [3–5].

The clinical diagnosis of PVO is difficult: half of patients are not febrile, and back pain, which is the most frequent symptom, is quite common in general population [5].

The low incidence and the nonspecific clinical presentation of the disease can contribute to empirical prescriptions of anti-inflammatory drugs, especially when patients have a back pain antecedent.

We studied the impact of these medications on diagnosis delay, clinical presentation at hospitalization, and prevalence of complications (Systemic Inflammatory Response Syndrome (SIRS), neurologic deficit, and positive blood culture) among patients with PVO.

## 2. Material and Methods

### 2.1. Study Settings

We performed a prospective study enrolling 79 patients with PVO. Patients were part of a multicenter, open-label, noninferiority, randomized, control trial studying antibiotic treatment duration in PVO [5]. Patients of our study were the first 79 patients included in this trial in the 2 main recruiting centers (University Hospital of Garches and University Hospital of Tours) from November 15, 2006, to November 15, 2010.

Inclusion criteria were the following: (1) clinical symptoms suggestive of PVO; (2) diagnosis of PVO assessed by MRI, CT scan, and/or bone scintigraphy; and (3) reliable microbiological identification defined as a positive bacterial culture of, at least, one deep sample (blood culture or vertebral biopsy). If the microorganism was a potential contaminant, 2 deep positive samples with a concordant identification were required.

Exclusion criteria were undocumented or nonpyogenic PVO or PVO on orthopedic device.

### 2.2. Ethical Committee

The French Data Protection Agency (CNIL) and the institutional review board of Versailles University Hospital (authorization number 06030) approved the protocol. The study was done in accordance with the ethical principles of the Declaration of Helsinki and the Guidelines for Good Clinical Practice. Written informed consent for participation in the trial was obtained from all patients.

### 2.3. Data Collection

Patient characteristics, clinical signs, radiological results, and microbiological identification were prospectively gathered. The C-reactive protein (CRP) dosages and positive blood culture were recorded on the first day of hospitalization.

Past medical history and anamnestic data since the beginning of symptoms were collected at admission in hospital: date of onset of symptoms and initial clinical presentation (fever defined as temperature >38°C and presence of pain). Nonsteroidal anti-inflammatory drugs (NSAIDs) and corticosteroids intake (without quantitative details) were recorded from the patient and general practitioner interviews.

### 2.4. Definitions

Diagnostic delay (DD) was defined as the time (day) from the first symptoms to diagnosis.

Chronical back pain was defined as pain lasting for more than 3 months. Rheumatoid arthritis, ankylosing spondylitis, and lupus were defined as inflammatory rheumatism.

Neoplasia history included solid or hematological current neoplasia. Neuropathy history included all the sensitive or motor neurological involvement (proved diabetic neuropathy, paraplegia, multiple sclerosis, etc.). Drugs abusers were active intravenous drug abusers. Minor neurocognitive impairment was a notion related by the general practitioner.

### 2.5. Objectives

The objectives were to determine the impact of anti-inflammatory drug intake on diagnosis delay, clinical presentation at hospitalization, incidence of complications (Systemic Inflammatory Response Syndrome (SIRS) and neurologic deficit) and cure rate among PVO cases.

### 2.6. Patient Groups

We divided the patients into 4 groups: group 1, patients without anti-inflammatory drug intake; group 2, patients who took anti-inflammatory drugs (NSAIDs or corticosteroids); group 3, patients who only used NSAIDs; and group 4, patients who only used corticosteroids.

### 2.7. Statistical Analysis

All continuous variables are presented as median and interquartile range, and the categorical variables are presented as frequencies.

First, univariate analysis was performed, studying correlations between NSAIDs, corticosteroids, any anti-inflammatory drugs intake, and diagnosis delay, clinical presentation at hospitalization, and incidence of complications of PVO in patients, using Student's *t*-test for continuous variables and Pearson's *χ* ^2^ or Fischer exact test for categorical variables.

Then, a multivariate analysis was performed with logistic models regression to study impact of NSAIDs, corticosteroids, and any anti-inflammatory drugs intake with different parameters of clinical significance as endpoints: diagnosis delay, fever at diagnosis, presence of SIRS, motor weakness, positive blood culture, and cure rate.

All reported probability values (*p* values) were based on two-sided tests, and a *p* value < 0.05 was considered statistically significant. All analyses were performed using the Statistical Package for Social Science (SPSS) version 17.0 (SPSS, Chicago, IL, USA).

## 3. Results

Overall, 79 patients were included; 71% were men. Mean age was 69 years old. Characteristics of study population are described in Table 1.

Of the 79 patients, 45 (57.0%) patients had neither NSAID nor corticosteroid intake, while 34 (43.0%) received anti-inflammatory drugs prior to PVO diagnosis, with 26 (32.9%) patients taking NSAIDs, 14 (17%) taking corticosteroids, and 6 (7.5%) using both.

PVO predominantly affected the lumbar and lumbosacral spine in over 68% of cases and in 24% of cases for the thoracic spine and in 20% of cases for the cervical spine. Multiple localizations were found in 17% of cases.

*Staphylococcus* spp. were nearly half of pathogens identified,* Streptococcus* spp. were identified in 22% of cases, and* Enterobacteriaceae* were identified in about 13% of cases.

The mean level of C-reactive protein (CRP) at diagnosis was 114 mg/L and 78% of patients had positive blood cultures.

Univariate analysis found no significant difference between the four study groups considering the characteristics of the population and of the PVO (localization and microbiological identification), diagnosis delay, motor weakness, CRP level and positive blood culture at diagnosis, and outcome (Table 1). The only significant difference found was that patients who took anti-inflammatory drugs had more painful symptoms and were less febrile, especially patients who only used NSAIDs.

Multivariate analysis found no correlation between any anti-inflammatory drug intake and fever, diagnosis delay, SIRS, motor weakness, positive blood culture, or cure rate.

## 4. Discussion

Our study population is similar to previous descriptions in literature, regarding age, localization of PVO, proportion of positive blood culture, and microorganisms involved [1–3, 5].

Despite easier access to MRI than in the 90s, diagnosis delay is still long: 30 days in median in our study.

NSAIDs are the most commonly prescribed painkillers for back pain, even when the pain's mechanical nature is still uncertain [6]. Thus, in the case of PVO where pain is the main suggestive symptom, this class of drugs could be misused and could modify its clinical presentation. Therefore, the diagnosis might be delayed and could be made at the late stage of neurological complications or severe sepsis. Furthermore, by its own anti-inflammatory action, it could be responsible for severe presentation.

However, our study did not find any statistically significant difference considering diagnosis delay, neurological complication, positive blood culture, or cure rate among patients with or without NSAIDs or corticosteroids consumption. We only noted that patients taking anti-inflammatory drugs felt more pain. Obviously, patients are more subject to using anti-inflammatory drugs in case of painful symptoms.

Thus, the prescription of anti-inflammatory drugs (mainly NSAID) to patients complaining from back pain will not delay the diagnosis of an eventual PVO, nor will it lead to a more severe clinical picture.

Moreover, we found that patients with any anti-inflammatory drugs intake, especially NSAIDs, were less febrile. Hence, physicians should suspect PVO in the presence of back pain, especially if this symptom is persistent and not well controlled by pain killers.

Several studies have shown that NSAIDs use could be significantly associated with a more severe presentation of bacterial infectious disease, with a reduced chemotactism of neutrophils shown in vitro [7–10]. According to experimental models it could be due to late diagnosis created by NSAIDs intake instead of their anti-inflammatory activity [11]. Also, the in vivo effect of NSAIDs on granulocyte function and cytokines is controversial. It has been described that ibuprofen increases endotoxin-induced TNF*α* and polymorphonuclear leukocyte degranulation in vivo [12].

Here, anti-inflammatory drugs seem not to be an aggravating circumstance.

This can be explained by the chronic characteristic of bone and joint infection, particularly PVO, and the usual slow replication of microorganism.

To our knowledge, our study is the first that studied the impact of anti-inflammatory drugs on the diagnosis delay and complications of PVO.

Although our study was conducted on a small number of patients and NSAIDs and corticosteroid intake were retrospectively collected and only qualitative, our results are reassuring. Ethically, no trial could be performed on this topic and we need large cohorts to confirm these preliminary data. Nevertheless, we still have to be precautious about prescribing NSAIDs or corticosteroids for back pain if PVO is not formally excluded.

## 5. Conclusion

Intake of anti-inflammatory drugs does not seem to have any significant impact on the diagnosis delay of PVO, the severity of its presentation, the incidence of its complications, and outcome. They could be prescribed for afebrile back pain, even if it is due to PVO. Further data are needed to confirm this trend due to our limited sample size and low statistical power.

## Figures and Tables

**Table 1:** Characteristics of patients presenting pyogenic vertebral osteomyelitis receiving nonsteroidal anti-inflammatory drugs (NSAIDs) versus patients receiving no NSAID.

Characteristics	Total (*n* = 79)	No anti-inflammatory drugs intake (*n* = 45)	Any anti-inflammatory drugs intake (*n* = 34)	*p* value	With NSAIDs intake (*n* = 26)	*p* value	With corticosteroids intake (*n* = 14)	*p* value
*Age*, years (median [IQR])	69 [53–78]	70 [53–77]	66 [52–78]	0.72	62 [50–75]	0.31	75 [53–78]	0.38
*Gender, male*,* n* (%)	55 (71)	34 (76)	21 (64)	0.25	17 (68)	0.50	8 (57)	0.18
*Antecedent*,* n* (%)								
Chronical back pain	22 (28)	13 (29)	9 (27)	0.81	7 (27)	0.86	4 (29)	1.00
Inflammatory rheumatism	4 (5)	1 (2)	5 (15)	0.72	2 (8)	0.55	2 (14)	0.14
Neoplasia	16 (20)	10 (22)	3 (9)	0.31	5 (19)	0.77	2 (14)	0.71
Diabetes mellitus	13 (17)	13 (29)	6 (18)	0.62	0	*∗∗∗*	0	*∗∗∗*
Neuropathy	5 (6)	3 (7)	0	*∗∗∗*	0	*∗∗∗*	2 (14)	0.58
Drugs abusers	4 (5)	2 (4)	2 (6)	1.00	2 (8)	0.62	0	*∗∗∗*
Mild neurocognitive impairment	2 (3)	0	2 (6)	1.00	2 (8)	*∗∗∗*	0	*∗∗∗*
*Diagnosis delay*, days (median [IQR])	30 [9–60]	27 [9–56]	32 [14–67]	0.28	40 [17–75]	0.10	26 [10–63]	0.79
*Initial presentation*,* n* (%)								
Initial pain	67 (87)	34 (79)	33 (97)	0.04	25 (96)	0.08	14 (100)	*∗∗∗*
Initial temperature >38°C	43 (57)	30 (70)	13 (39)	0.01	8 (32)	0.002	6 (43)	0.07
*Topography*								
Plurifocal	13 (17)	8 (18)	5 (15)	0.72	3 (12)	0.74	3 (21)	0.71
Epiduritis	30 (41)	17 (42)	13 (39)	0.86	11 (42)	0.95	4 (31)	0.54
Cervical localization	16 (20)	10 (22)	6 (18)	0.62	5 (19)	0.77	2 (14)	0.52
Thoracic localization	19 (24)	11 (24)	8 (24)	0.93	8 (31)	0.56	2 (14)	0.71
Lumbar localization	42 (53)	22 (49)	20 (59)	0.38	12 (46)	0.82	12 (86)	0.03
Sacral localization	12 (15)	7 (16)	5 (15)	0.92	5 (19)	0.69	1 (7)	0.67
*Microbiology*								
MSSA	27 (34)	16 (36)	11 (32)	0.77	8 (31)	0.68	6 (43)	0.62
CoNS	12 (15)	8 (18)	4 (12)	0.54	4 (15)	1.00	1 (7)	0.67
MRSA	1 (1)	1 (2)	0	*∗∗∗*	0	*∗∗∗*	0	*∗∗∗*
*Streptococcus* non-*Enterococcus*	17 (22)	8 (18)	9 (27)	0.35	6 (23)	0.59	5 (36)	0.16
*Enterococcus* spp.	3 (4)	1 (2)	2 (6)	0.57	2 (8)	0.55	0	*∗∗∗*
*Escherichia coli*	8 (10)	4 (9)	4 (12)	0.72	3 (12)	0.70	1 (7)	1.00
*Klebsiella pneumoniae*	2 (3)	1 (2)	1 (3)	1.00	0	*∗∗∗*	1 (7)	0.42
Others	11 (14)	7 (16)	4 (12)	0.75	3 (12)	0.74	1 (7)	0.67
*In-hospital presentation*								
Pain	72 (91)	39 (87)	33 (97)	0.23	25 (96)	0.25	14 (100)	*∗∗∗*
Fever	41 (53)	26 (61)	15 (44)	0.15	11 (42)	0.14	5 (36)	0.11
SIRS	22 (28)	16 (36)	6 (18)	0.08	3 (12)	0.05	3 (21)	0.51
Motor weakness	10 (13)	6 (14)	4 (12)	1.00	2 (8)	0.70	2 (15)	1.00
*Biological*								
CRP, mean (mg/L)	114 [62–229]	108 [68–240]	125 [53–190]	0.42	80 [44–182]	0.25	111 [62–207]	0.59
Positive blood culture (*n*, %)	61 (78)	32 (73)	29 (85)	0.18	21 (81)	0.45	14 (100)	*∗∗∗*
*Outcome*								
Cure rate at 1 year	72 (72/79)	41 (41/45)	31 (31/34)	1.00	24 (24/26)	1.00	13 (13/14)	1.00

NSAIDs: nonsteroidal anti-inflammatory drugs; IQR: interquartile range; MSSA: methicillin-susceptible *Staphylococcus aureus*; CoNS: coagulase negative staphylococci; MRSA: methicillin-resistant *Staphylococcus aureus*; SIRS: systemic inflammatory response syndrome; CRP: C-reactive protein; *∗∗∗*: not applicable.

## References

[1] Grammatico L., Baron S., Rusch E. (2008). Epidemiology of vertebral osteomyelitis (VO) in France: analysis of hospital-discharge data 2002-2003. *Epidemiology and Infection*.

[2] Kehrer M., Pedersen C., Jensen T. G., Lassen A. T. (2014). Increasing incidence of pyogenic spondylodiscitis: a 14-year population-based study. *Journal of Infection*.

[3] Murillo O., Roset A., Sobrino B. (2014). Streptococcal vertebral osteomyelitis: multiple faces of the same disease. *Clinical Microbiology and Infection*.

[4] SPILF (2007). Primary infectious spondylitis and following intradiscal procedure, without prothesis. Recommendations. *Medecine et Maladies Infectieuses*.

[5] Bernard L., Dinh A., Ghout I. (2015). Antibiotic treatment for 6 weeks versus 12 weeks in patients with pyogenic vertebral osteomyelitis: an open-label, non-inferiority, randomised, controlled trial. *The Lancet*.

[6] Roelofs P. D., Deyo R. A., Koes B. W., Scholten R. J., van Tulder M. W. (2008). Non-steroidal anti-inflammatory drugs for low back pain. *Cochrane Database of Systematic Reviews*.

[7] Bertolotto M., Contini P., Ottonello L., Pende A., Dallegri F., Montecucco F. (2014). Neutrophil migration towards C5a and CXCL8 is prevented by non-steroidal anti-inflammatory drugs via inhibition of different pathways. *British Journal of Pharmacology*.

[8] Voiriot G., Dury S., Parrot A., Mayaud C., Fartoukh M. (2011). Nonsteroidal antiinflammatory drugs may affect the presentation and course of community-acquired pneumonia. *Chest*.

[9] François P., Desrumaux A., Cans C., Pin I., Pavese P., Labarère J. (2010). Prevalence and risk factors of suppurative complications in children with pneumonia. *Acta Paediatrica*.

[10] Souyri C., Olivier P., Grolleau S., Lapeyre-Mestre M. (2008). Severe necrotizing soft-tissue infections and nonsteroidal anti-inflammatory drugs. *Clinical and Experimental Dermatology*.

[11] Guibal F., Muffat-Joly M., Terris B., Garry L., Morel P., Carbon C. (1998). Effects of diclofenac on experimental streptococcal necrotizing fasciitis (NF) in rabbit. *Archives of Dermatological Research*.

[12] Spinas G. A., Bloesch D., Keller U., Zimmerli W., Cammisuli S. (1991). Pretreatment with ibuprofen augments circulating tumor necrosis factor-*α*, interleukin-6, and elastase during acute endotoxinemia. *Journal of Infectious Diseases*.

